# On the Potential Role of Dietary Lysine as a Contributing Factor in Development of Liver Abscesses in Cattle

**DOI:** 10.3389/fvets.2020.576647

**Published:** 2020-10-02

**Authors:** Vanessa Aguiar Veloso, James S. Drouillard

**Affiliations:** Department of Animal Sciences and Industry, Kansas State University, Manhattan, KS, United States

**Keywords:** liver abscess, *Fusobacterium necrophorum*, cattle, lysine, feedlot

## Abstract

Liver abscessation is an important metabolic disorder that commonly afflicts cattle consuming cereal-based, high-concentrate diets. Economic ramifications of liver abscessation are substantial, and include liver condemnation, decreased body weight gain, poorer efficiency of feed utilization, reduced carcass yield, and impairments in operational efficiency of commercial abattoirs. The etiological agent most commonly associated with liver abscesses is *Fusobacterium necrophorum*, which is an anaerobic, Gram-negative, nonmotile, nonsporulating, and rod-shaped (pleomorphic) bacterium. *Fusobacterium necrophorum* is one of the major proteolytic species of bacteria in the rumen, and it is believed to have a major role in degradation of dietary lysine. Herein we describe interactions between lysine and *F*. *necrophorum*, and the potential role of dietary lysine as an enabling factor in the development of liver abscesses in cattle.

## Introduction

Aggressive feeding programs, such as those used by North American feedlot operations, often are associated with increased incidence of liver abscesses in cattle at slaughter (1). Data from Brown et al. (2) revealed that liver abscesses decreased daily weight gain and efficiency of feed utilization in feedlot cattle. When comparing slaughtered cattle with severe liver abscesses vs. healthy cattle or animals with minor abscesses, Brown et al. (3) reported that severe abscesses decrease gain and resulted in poorer dressing percentages compared to animals with healthy livers. The rate of liver condemnations in U.S. cattle has been estimated to be 44.6%, with most condemnations being due to liver abscessation (4). A variety of dietary characteristics have been studied and associated with incidence of liver abscesses in feedlot cattle. Generally, the incidence is affected by amount and type of forage included in the finishing diets. Animals fed diets with greater proportions of roughage were observed to have lower incidence of liver abscesses (5). Utley et al. (6) observed that physical characteristics of the forage tended to impact incidence of liver abscesses. Additionally, grain type also was described to affect incidence of liver abscess (7), presumably as a result of differences in rate of ruminal digestion. Incidence of liver abscesses typically is greater in feedlot steers than in heifers, which likely is due to differences in feed intake between heifers and steers (8). Holstein feedlot steers, which normally are fed for longer periods compared to beef breeds, also have greater incidence of abscessed livers compared to slaughtered beef breeds (8).

Liver abscesses are a major cause of liver condemnations in packing plants, and the economic impact of liver condemnations is heavily influenced by suboptimal animal performance and decreased carcass yield compared to healthy cattle (1, 9), as well as decreases in line speed at commercial abattoirs. Brown and Lawrence (10) estimated an annual economic loss of $7,007,797 to the beef industry due to liver abscessation in cattle. Severely abscessed livers are frequently found adhered to the diaphragm and/or abdominal organs, which results in substantial losses due to trimming of compromised areas of the carcass. Carcass trimmings reduce total hot carcass weight and are estimated to cause an opportunity loss of more than 25 million kg of carcass weight in US beef production (10). The estimated decrease in carcass value of an animal with a severely abscessed liver (i.e., livers with multiple large abscesses, livers adhered to abdominal organs and/or diaphragm, and livers with ruptured abscesses) range from $25.54 (10) to more than $52 (11).

Formation of liver abscesses in ruminants is regarded as a secondary sequelae to ruminal acidosis, which can result in ruminal lesions that allow pathogens to enter into the portal blood supply (12). Hence, the same factors that lead to development of ruminal acidosis and ruminitis, such as irregular bunk management and abrupt changes in amount or fermentability of the diet, can induce liver abscessation in cattle. Severe abscesses also can affect surrounding tissues, leading to increased carcass trim losses during processing and reduced carcass weight. Rupture of abscesses during processing can lead to contamination of the carcass, often requiring extensive trimming of the carcass and interruption of the slaughter chain flow (1).

## Liver Abscess in Feedlot Cattle

Liver abscesses are the primary liver abnormalities of feedlot cattle recorded at the time of slaughter (10). According to the National Beef Quality Audit-2016, 44.6% of livers were condemned at slaughter, and liver abscesses accounted for 46.4% of the total liver condemnations (4), thus affecting over 20% of the U.S. fed cattle population.

Liver abscesses have a significant economic impact on the feedlot cattle industry, which varies according to the severity of liver abscesses (i.e., severely abscessed livers may be found adhered to abdominal organs and diaphragm resulting in carcass trimming). Based on the number and size of abscesses, liver abscesses are scored on a scale of 1 to 3, as mild, moderate, and severe, respectively [or as A-, A, and A+; (3)]. Brown and Lawrence (10) estimated a decrease in carcass returns of US$20 to US$80 as the opportunity value lost due to liver abscessation in the beef industry per animal.

Although it is well stablished that *F. necrophorum* is the primary etiological agent of liver abscessation in feedlot cattle, bacteriologic studies performed in abscessed livers have shown that other microbes can be involved (7). In most cases, the second most frequent pathogen isolated from liver abscesses is *Trueperella pyogenes* (previously identified as *Arcanobacterium pyogenes*; 4).

*Trueperella pyogenes* is a Gram-positive, pleomorphic, rod-shaped bacterium, and a facultative anaerobe. Generally, the occurrence of *T. pyogenes* in hepatic abscesses range from 2 to 40% (8). Furthermore, occurrence of *T. pyogenes* in hepatic abscesses is almost always associated with *F. necrophorum* [i.e., isolated from 100% of the hepatic abscesses; (8)]. Such observations lead other authors to speculate the existence of a pathogenic synergism between the two species (13), which could potentially occur either during initial establishment of *F. necrophorum* in the ruminal wall or in the liver parenchyma. It has been hypothesized that *T. pyogenes* utilizes oxygen, creating an anaerobic environment that is optimal for multiplication of *F. necrophorum*. Furthermore, a number of other anaerobic and facultative bacteria have been isolated from hepatic abscesses, including *Bacteriodes* spp., *Clostridium* spp., *Escherichia coli, Klebsiella* spp., *Enterobacter* spp., *Mobilincus* spp., *Pasteurella* spp., *Peptostreptococcus* spp., *Porphyromonas* spp., *Prevotella* spp., *Propionibacterium* spp., *Staphylococcus* spp., *Streptococcus* spp., *Salmonella enterica*, and others (8).

## Fusobacterium Necrophorum

*Fusobacterium necrophorum* has been identified as the primary etiologic agent of liver abscesses in feedlot cattle (7). It is an anaerobic Gram-negative, nonmotile, nonsporulating, and rod-shaped (pleomorphic) bacterium. Members of the genera *Fusobacterium* are the most common anaerobes identified in animals with infectious pyonecrotic processes, where *F. necrophorum* is the most commonly isolated specie (14).

In the rumen, *F. necrophorum* utilizes lactate as its primary energy substrate (13). Because *F. necrophorum* is a lactate-fermenting bacterium, its concentrations in the rumen tended to be 10-fold greater in grain-fed cattle than cattle fed forage-based diets (13). Russell (15) also observed that *Fusobacterium spp*. isolated from the rumen could utilize lysine as an energy source for growth. In fact, the strains isolated from dairy cattle fed timothy hay or a commercial dairy ration degraded lysine rapidly (15). Amino acids are degraded in the rumen by a group of bacteria generally classified as hyper-ammonia producers (16). Attwood et al. (17) identified a strain of *F. necrophorum* isolated from the rumen that was hyper-ammonia producing. Thereafter, Russel (15) described *F. necrophorum's* great capacity for degrading lysine. Those observations led subsequent authors to hypothesize that *F. necrophorum* may be the major bacteria involved in lysine degradation within the rumen. Elwakeel et al. (18) conducted *in vitro* studies to characterize lysine degradation by ruminal *F. necrophorum* using seven ruminal isolates that were grown on lactate or lysine as the primary energy sources. Both sub-species of ruminal *F. necrophorum* (*necrophorum* and *fundiliforme*) were described to use lysine as their primary source of carbon and energy.

Aguiar Veloso (19) conducted a study in which a ruminally-protected form of lysine was fed at 4 different levels (0, 20, 40, or 60 g/animal daily) to finishing beef steers during the final 42 days of the feedlot finishing period, and observed a dose-dependent increase in liver abscesses (*P* < 0.04) as the amount of supplemental lysine was increased in the diet. Additionally, supplementing lysine tended (*P* = 0.07) to increase severity of liver abscesses. The mechanism by which lysine supplementation stimulated formation of liver abscesses in finishing cattle was not readily evident, but the observation raises questions concerning the possible role of lysine in stimulating proliferation and (or) virulence of *F. necrophorum* within the rumen, post-ruminal gastrointestinal tract, or in the liver.

## Prophylaxis

The control of liver abscesses in feedlot cattle has been heavily dependent on the use of antimicrobial compounds. Antibacterial drugs are largely incorporated in feedlot diets to improve overall animal health and feed conversion (20); however, the use of antibiotics in livestock production has become a matter of concern. Although there is some concern with regard to the presence of antibiotic residues in meat, generally this would be considered minor when compared with the issue of selection and amplification of antibiotic resistant strains of bacteria. Extensive use of sub-therapeutic doses of antimicrobial agents as growth promotants constitutes selective pressure that can induce local bacteria populations to develop resistance to antibiotic agents. The emergence of microbial resistance may compromise therapeutic use of antibiotics in various ways, such as: (1) selection of microbes that are direct human pathogens; (2) selection of resistant zoonotic microorganisms (i.e., pathogenic to animals and humans); (3) resistance determinants may be selected in a bacterium that is a member of the commensal flora that subsequently can become pathogenic to humans, animals, or both (21). Furthermore, to reduce the risk of selecting resistant bacteria, the use of antibiotics must be restricted, and the most reasonable area for reducing the use of antibiotics is their use as growth promoters in food animals. In June of 2015, the U.S. Food and Drug Administration (FDA) announced their final rule aimed at restricting access to “medically important” drugs and prohibiting their use as growth promotants. In January 2017, the FDA Center for Veterinary Medicine (CVM) and animal drug manufacturers completed the voluntary transition of all medically important antimicrobial drugs used in or on animal feed, from over-the-counter to Veterinary Feed Directive marketing status, thus requiring a veterinary prescription for their use.

A 10-year net change for retail beef supply after removing preventative liver abscess controls was estimated by Olvera (22), who predicted a reduction in total quantities of 6.31% over that timeframe, corresponding to a loss of 510 million kilograms of beef. If causational factors of liver abscess were identified, alternative preventive or curative methods could perhaps be more quickly identified and compared. Mir et al. (23) observed that sunflower seed supplementation to finishing steers reduced incidence and severity of liver abscesses. Others described a quadratic decrease in incidence of liver abscesses when any amount of dried full-fat corn germ was included in finishing cattle diets (24). The authors attributed decreases in liver abscesses to the decrease in starch intake and to changes in feed intake patterns caused by full-fat corn germ supplementation. The full-fat corn germ used in their research was, however, dried and thus was subject to formation of Maillard reactions end-products, including the brown melanoidin pigments (25). It is worthy to note that melanoidins interfere with normal proteolytic activity (26), and also are known to have activity against specific microbial populations (27), thus it is conceivable that decreases in liver abscessation were a consequence of indirect effects on *Fusobacteria sp*. through suppression of its proteolytic activity.

Although there is an established relationship between highly fermentable diets and hepatic abscessation in ruminants, the impact of other dietary characteristics and ingredient profiles have not been fully explored. Aguiar Veloso (19) initially described a relationship between lysine supplementation and liver abscessation in feedlot steers; however, the author failed to replicate the observation in a second study. To the best of our knowledge there are no studies that investigate impact of dietary protein concentration on incidence and severity of liver abscessation in cattle. When Prior et al. (28) fed diets with low, medium, and high levels of energy and protein, incidence of liver abscessation was reported with respect to dietary energy levels but impact of dietary protein concentration was not elucidated. Thus, further studies are needed to determine the impact of dietary protein level, as well as other nutrients, on the incidence and severity of hepatic abscessation in cattle.

## Conclusions and Discussion

In summary, from the current knowledge, we infer that dietary lysine may play an important role in the formation of liver abscesses in cattle fed high-concentrate diets. Furthermore, we speculate that this may be the result of its effects on proliferation or virulence of *Fusobacterium necrophorum*, the predominant etiological agent associated with abscessed livers. It is unknown if this effect occurs within the rumen, post-ruminal gastrointestinal tract, or liver. A more complete understanding of the role of lysine in formation of liver abscesses could be important in identifying alternative, non-antibiotic mitigation methods.

## Author Contributions

VAV and JD were responsible for conceptualization and writing, review, and editing. Both authors have approved this manuscript for publication.

## Conflict of Interest

The authors declare that the research was conducted in the absence of any commercial or financial relationships that could be construed as a potential conflict of interest.
